# Development and Validation of a Nomogram‐Based Risk Stratification Model for Moderate‐to‐High Frequency Workplace Violence Among Chinese Pediatric Nurses: A Cross‐Sectional Study

**DOI:** 10.1155/jonm/6562210

**Published:** 2026-09-24

**Authors:** Xuemei Ding, Huichen Zhang, Mengting Liu, Xiaoyu Pan, Qingmei Ding, Yuhong Zhang, Xiaoli Zhang

**Affiliations:** ^1^ School of Nursing, Shandong Medical and Pharmaceutical University, Yantai 264003, China; ^2^ School of Health Management, Shandong Medical and Pharmaceutical University, Yantai 264003, China; ^3^ Department of Pediatrics, Yantai Yuhuangding Hospital, Yantai 264000, China, ytyhdyy.com; ^4^ Department of Gastroenterology, Dongying People’s Hospital, Dongying 257000, China, dysrmyy.com/

**Keywords:** nomogram, pediatric nurses, risk stratification, synthetic minority over-sampling technique, workplace violence

## Abstract

**Background:**

Workplace violence (WPV) against pediatric nurses remains a major occupational concern, but practical tools for concurrent risk stratification are limited.

**Objective:**

To develop and validate a nomogram‐based model for concurrent risk stratification of moderate‐to‐high frequency WPV among Chinese pediatric nurses, and to assess class imbalance using the synthetic minority over‐sampling technique (SMOTE) as a complementary analysis.

**Methods:**

This multicenter cross‐sectional study included 592 pediatric nurses from 10 tertiary hospitals in Shandong, China. Electronic questionnaires assessed 24 variables across individual, interpersonal, organizational, and policy levels. Participants were randomly divided into training and test sets at a 7:3 ratio. Primary and complementary logistic regression models were developed using the original and SMOTE‐resampled training sets, respectively. Models were evaluated using discrimination, calibration, decision curve analysis, risk stratification, and sensitivity analyses.

**Results:**

In the primary model, hospital level, nurse–child–caregiver communication conflicts, tolerance of violence, occupational stress, and burnout were independently associated with moderate‐to‐high frequency WPV. The model showed stable discrimination, with area under the curve (AUC) values of 0.794 in the training set and 0.808 in the test set, and acceptable calibration in both datasets. The optimal cutoff was 0.195, which effectively stratified nurses into low‐ and high‐risk groups in both datasets (*p* < 0.001). In the SMOTE‐based analysis, fair or poor health status, delayed hospital attitudes to WPV, and blaming employees additionally became significant. This model achieved comparable discrimination, with AUC values of 0.814 and 0.805 in the resampled training and original test sets, respectively, and higher sensitivity, but poor calibration in the test set, particularly at higher predicted probabilities.

**Conclusions:**

The primary nomogram may serve as a practical decision‐support tool for concurrent WPV risk stratification, whereas the SMOTE‐based model should be regarded as a complementary analysis. These findings may help nursing managers identify nurses who currently require prioritized prevention and support.

## 1. Introduction

Workplace violence (WPV) refers to abusive, threatening, or assaultive behavior experienced by employees during occupational activities, which poses a serious threat to their physical and mental health as well as occupational safety. WPV is highly prevalent in healthcare settings worldwide. A meta‐analysis reported that annual exposure rates among healthcare workers range from 25.0% to 78.9% [1]. In China, WPV is similarly common, with an estimated prevalence of approximately 71.0%, and nurses are particularly vulnerable [2]. Liu et al. found that nurses had a 3.66‐fold higher risk of WPV than other medical staff [2]. In certain high‐stress departments, exposure rates may reach 85.0% [3], and a considerable proportion of incidents occur at moderate‐to‐high frequency [4, 5]. Furthermore, the circumstances, perpetrators, and patterns of WPV may differ across clinical populations, suggesting that findings from general healthcare settings may not be directly generalizable to specific populations such as pediatric nurses.

Pediatric nurses warrant specific attention, as WPV in pediatric settings may arise within a distinctive family‐centered care context involving the nurse, the child, and the child’s caregivers [6]. Several potentially interacting factors may increase the likelihood of conflict within this context. First, unlike many adult patients, children may have limited ability to describe their symptoms, understand clinical procedures, or participate independently in care decisions. Nurses, therefore, often rely on caregivers to communicate clinical information about the child [7]. However, this information may be incomplete or interpreted differently, increasing the potential for misunderstanding [8]. Second, caregivers’ anxiety or fear regarding the child’s condition, together with unmet expectations concerning treatment or recovery, may contribute to emotional tension and dissatisfaction [9, 10]. Third, technically demanding procedures, such as venipuncture and blood sampling, may heighten caregiver distress or dissatisfaction, particularly when repeated attempts are required or when the child cries, resists, or experiences distress [11]. Collectively, the interplay between caregiver involvement and these communication, emotional, and procedural challenges may create circumstances conducive to WPV against pediatric nurses. Consistent with this, qualitative interviews identified unmet family expectations, misunderstandings regarding treatments and procedures, and communication difficulties as circumstances associated with verbal violence [10]. A cross‐sectional study of 317 nurses at the Vietnam National Children’s Hospital found that 72.7% had experienced WPV, with caregivers accounting for 85% of the perpetrators [12]. Similarly, a study of 528 pediatric nurses reported that all participants had experienced at least one form of verbal threat during the preceding three months, with parents or visitors identified as the most frequent perpetrators (75.9%) [13]. These findings underscore the prominent role of interactions with caregivers in WPV against pediatric nurses. Beyond overall prevalence, the frequency of WPV merits particular attention because prevalence estimates alone cannot distinguish isolated incidents from recurrent exposure. In the qualitative study of pediatric emergency nurses, more than half of the participants reported experiencing violence approximately once a month [10]. A study conducted in Zhengzhou found that 21.1% of pediatric healthcare workers reported moderate‐frequency WPV and 4.8% reported high‐frequency WPV [5]. Although the study included pediatric healthcare workers other than nurses, its findings provide contextual evidence that moderate‐to‐high frequency WPV occurs in pediatric settings, a phenomenon that has rarely been quantified specifically among pediatric nurses. This gap limits the identification of pediatric nurses recently at risk of moderate‐to‐high frequency exposure and constrains the development of targeted interventions for this high‐risk group.

WPV is associated with adverse outcomes among pediatric nurses and may compromise healthcare delivery. A phenomenological study found that pediatric nurses reported experiencing a wide range of adverse psychological responses, including stress, frustration, insecurity, fear, anger, and depressive feelings, following exposure to WPV [14]. Similarly, pediatric nurses and technicians who had experienced WPV reported significantly higher overall stress levels and a greater burden of somatic symptoms, including fatigue, headaches, and sleep difficulties, than those without such exposure [15]. WPV was also associated with posttraumatic stress disorder symptoms, with psychological resilience and emotion regulation partially mediating this association and accounting for 30.81% and 39.89% of the total effect, respectively [16]. Qualitative findings from pediatric intensive care nurses further linked psychological distress to interpersonal tension and WPV, suggesting a potentially reinforcing interplay between WPV and psychological distress [17]. Beyond these physical and psychological consequences, WPV has also been found to adversely affect patient safety and the overall quality of care [17]. Consistent with this finding, WPV was negatively correlated with nursing job performance (*r* = −0.694, *p* < 0.001) [18]. Additionally, WPV may be perceived as inevitable or part of the job, reflecting its normalization within the workplace and potentially hindering reporting and effective responses to unsafe working conditions [19, 20]. Addressing this issue, therefore, may require timely stratification of nurses according to their current risk status, enabling targeted interventions.

Existing approaches to WPV assessment serve different populations and purposes. First, exposure‐surveillance instruments, such as the WPV in the Health Sector Country Case Study Questionnaire, are administered to healthcare workers to retrospectively document the occurrence and type of WPV, perpetrators, circumstances, and other related details [21]. These instruments are essential for estimating prevalence and characterizing the burden of WPV, but they directly measure retrospectively reported exposure rather than integrating multilevel characteristics into an individualized probability of belonging to a predefined frequency group. Second, patient‐focused violence risk assessment tools, including the Brøset Violence Checklist, are designed to identify patients at risk of imminent aggression [22]. Because these tools assess potentially aggressive patients using observable behaviors, they serve a different purpose from the concurrent classification of nurses according to their recent exposure status.

Models for classifying or predicting healthcare workers’ WPV exposure have also emerged. Airaksinen et al. used least absolute shrinkage and selection operator regression to predict an increase in violence frequency among registered nurses. The final model incorporated work‐unit characteristics such as staffing composition, strenuous work effort, and influence over shift arrangements, and achieved an area under the curve (AUC) of 0.67 [23]. Using a random forest, Havaei et al. examined 13 psychosocial workplace factors and identified deficits in physical safety, workload management, and psychological protection as the most influential domains associated with type II WPV among nurses [24]. An interpretable machine‐learning study among Chinese junior nurses incorporated demographic, nursing‐related, and work‐related factors, including monthly income, job satisfaction, and number of patients [25]. Among Chinese emergency nurses, unsupervised clustering followed by a multilayer perceptron model used 15 demographic and occupational characteristics, including work experience, night‐shift frequency, and income, to identify the profile with the highest WPV scores (AUC = 0.628) [26]. These studies demonstrate the feasibility of using statistical and machine‐learning approaches to model healthcare workers’ WPV exposure. However, important gaps remain. First, existing models were developed primarily among registered nurses, junior nurses, emergency nurses, or broader healthcare‐worker populations, and their applicability to pediatric nurses remains uncertain. Second, the outcomes have ranged from any prior WPV exposure and subsequent changes in exposure frequency to data‐driven profiles partly defined by WPV scores. Few studies, however, have focused on a prespecified outcome of recent moderate‐to‐high frequency WPV exposure. Third, previous studies have incorporated variables across multiple domains, including demographic and occupational characteristics, work‐unit conditions, and psychosocial workplace factors. Nevertheless, the relevance of these factors remains unclear in pediatric nursing, where WPV occurs within a distinctive nurse–child–caregiver context. Although pediatric‐specific circumstances, such as caregiver‐mediated communication [10], have been discussed as potential associated factors of WPV, they have rarely been considered in previous modeling studies. In addition, WPV‐related policy variables have not been represented consistently across existing models. Finally, a recent systematic review concluded that existing models have limited clinical utility [27]. Accordingly, there is a need for an interpretable and practically applicable model that integrates multilevel characteristics to classify recent moderate‐to‐high frequency WPV specifically among pediatric nurses.

To provide a theoretical basis for addressing these gaps, the social ecological model (SEM) offers a useful conceptual perspective. The SEM conceptualizes violence as being influenced by factors operating across the individual, relational, organizational or community, and societal or policy levels [25, 28]. In the present study, these levels were operationalized as four domains: individual characteristics, interpersonal relationships, organizational environments, and policy support. This multilevel perspective may be particularly relevant to pediatric nursing, where caregiver involvement creates distinctive interpersonal dynamics that interact with nurse‐related and healthcare‐system factors [10]. Accordingly, organizing candidate variables across these four domains may provide a more comprehensive representation of the factors associated with pediatric nurses’ recent WPV exposure than examining any single domain in isolation.

Given the need for such a model, a nomogram may offer a suitable graphical format. By translating regression coefficients into a visual, point‐based scoring system, nomograms facilitate individualized classification and interpretation without requiring direct interpretation of regression coefficients or complex model outputs. For example, nomograms have been used for risk stratification of overactive bladder among female nursing staff [29] and for hierarchical management of high burnout risk among nurses [30]. A recent cross‐sectional study also developed a nomogram for compassion fatigue among emergency department nurses using demographic, occupational, and lifestyle‐related variables and reported good calibration and discrimination [31]. Although these outcomes differ from WPV, these studies demonstrate the feasibility of presenting multivariable occupational‐health information in an accessible and interpretable graphical format. Accordingly, a nomogram may provide a feasible approach to estimating an individual pediatric nurse’s probability of membership in a predefined recent moderate‐to‐high frequency WPV exposure group.

Guided by the SEM, this study aimed to identify multilevel factors associated with reported moderate‐to‐high frequency WPV exposure during the preceding 12 months among pediatric nurses in China. Based on original data, we developed and validated a nomogram‐based model for concurrent risk stratification. To account for class imbalance, we also conducted a complementary analysis using the synthetic minority over‐sampling technique (SMOTE) and compared its influence on variable selection and model performance. The models may support nursing managers in identifying nurses with a higher likelihood of recent moderate‐to‐high frequency WPV exposure and in developing targeted intervention strategies. These tools may also contribute to efforts to reduce WPV, protect pediatric nurses’ occupational safety and well‐being, and improve the quality of pediatric care.

The study proposes the following hypotheses:

• H_1_: Significant differences exist across individual characteristics, interpersonal relationships, organizational environments, and policy support between pediatric nurses with and without moderate‐to‐high frequency WPV exposure.
• H_2_: Moderate‐to‐high frequency WPV exposure is significantly associated with higher levels of occupational stress, burnout, and turnover intention among pediatric nurses.
• H_3_: The primary nomogram developed from multidimensional factors demonstrates acceptable performance for concurrent risk stratification of moderate‐to‐high frequency WPV among pediatric nurses. The SMOTE‐based model serves as a complementary analysis to assess the influence of class imbalance.

## 2. Materials and Methods

### 2.1. Study Design

This study employed a multicenter cross‐sectional survey that included a retrospective assessment of WPV over the preceding 12 months and was conducted from February 4 to March 2, 2024, in Shandong Province, China. As one of the most populous regions in China, Shandong has relatively abundant pediatric medical resources, with the number of registered pediatric nurses ranking among the highest in the country. Therefore, it was considered an appropriate setting for recruiting pediatric nurses from multiple tertiary hospitals. The study was reported following the Transparent Reporting of a Multivariable Prediction Model for Individual Prognosis or Diagnosis–Artificial Intelligence (TRIPOD + AI) statement (Supporting File 1).

### 2.2. Participants

A multistage sampling strategy was used to recruit study participants. First, Shandong Province was geographically divided into five regions: eastern, western, southern, northern, and central. Second, two tertiary hospitals were randomly selected from each region using a computer‐generated random number table. Finally, all pediatric inpatient wards in each selected hospital were treated as clusters, and all registered nurses meeting the inclusion criteria and working full‐time during the survey period were invited. Inclusion criteria included (a) registered nurses holding a valid People’s Republic of China Nurse Practice Certificate, (b) at least one year of clinical experience in pediatric nursing, and (c) provided consent for voluntary participation. Exclusion criteria included (a) nonpermanent nurses, such as interns or trainees, and (b) absence during the survey period due to maternity leave, sick leave, or long‐term leave. The study recruited 620 pediatric nurses who met the eligibility criteria. Data were collected using a structured electronic questionnaire, and the final analytic sample was determined after quality control procedures.

### 2.3. Data Collection

A pretest was conducted with 30 registered pediatric nurses from a nonparticipating hospital in Yantai City, Shandong Province, to assess the clarity, comprehensibility, acceptability, and potential ambiguity of the questionnaire. Based on feedback, the wording, options, and instructions were optimized to enhance clarity. Centralized training was conducted for the managers of pediatric nursing units at participating hospitals, covering the study background, objectives, and procedures; questionnaire guidelines and key definitions; data confidentiality measures; and investigator responsibilities, including informed consent procedures and neutral clarification methods. Unit managers provided detailed explanations of the study information to potential participants, including voluntary participation and confidentiality. They acted only as study contact persons and were instructed not to influence participation, monitor individual completion, or access individual responses. Electronic informed consent forms and unique questionnaire links were distributed via internal channels such as WeChat groups. Prior to accessing the questionnaire, participants were required to read the electronic informed consent form and confirm their willingness to participate. Participants were advised to complete the questionnaire during work breaks in a quiet area. The questionnaire took approximately 10 to 15 min to complete. Participation was anonymous and voluntary, and nurses could decline or withdraw at any time without penalty or employment‐related consequences. Throughout the data collection process, the research team monitored data quality, tracked submission progress only at the aggregate level through the Wenjuanxing platform (https://www.wjx.cn/), and maintained ongoing communication to address emerging issues and ensure data completeness.

### 2.4. Outcome

The Workplace Violence Frequency Measurement Scale (WVS) developed by Wang [32] was used to assess WPV exposure. Having undergone cross‐cultural adaptation, this tool has demonstrated good reliability and validity in measuring WPV prevalence among Chinese nursing staff. The scale employs a one‐year retrospective survey period. It covers five categories of violent behavior: physical assault, emotional abuse, threats and intimidation, verbal sexual harassment, and physical sexual harassment. Items are scored on a 4‐point Likert scale (0 = *never occurred*, 1 = *once*, 2 = *2–3 times*, and 3 = ≥ *4 times*). The total score, calculated by summing all items, is categorized as follows: zero frequency (0 points), low frequency (1–5 points), moderate frequency (6–10 points), or high frequency (11–15 points). In this study, a total score of ≥ 6 was defined as moderate‐to‐high frequency WPV. Cronbach’s α was 0.771, indicating acceptable reliability.

### 2.5. Candidate Variables

Guided by the SEM and considering patient privacy concerns and data accessibility, this study established a four‐level candidate variable framework for risk stratification, comprising individual characteristics, interpersonal relationships, organizational environment, and policy support. Within this framework, candidate variables were selected through a two‐stage process. First, a preliminary variable pool was developed based on a review of previous WPV studies among nurses, with particular attention to evidence from pediatric healthcare settings and China [9, 10, 33–35]. Second, this preliminary pool was evaluated through a structured expert consultation conducted through a panel meeting. The twelve‐member panel included three experts in pediatric clinical nursing, three in nursing management, two in occupational health, two in hospital quality management, and two in clinician–patient relationship research. All experts held senior professional titles and had more than 10 years of relevant professional experience. Before the meeting, the experts independently reviewed the preliminary variable list and supporting literature. During the meeting, each variable was evaluated for theoretical and empirical support, conceptual clarity, measurement feasibility, potential overlap, and consistency with the SEM. The experts then voted independently, with consensus predefined as at least 80% agreement. Variables were retained, revised, merged, or excluded based on expert input. Variables that did not reach consensus in the first round were further discussed and subjected to a second vote, after which the final candidate variable pool was determined.

Individual characteristics were categorized into demographic variables, including age, gender, marital status, highest education level, personality type, and self‐perceived health status [33, 35, 36]; occupational psychological variables, including occupational stress and burnout [37, 38]; and behavioral variables, including willingness to attend WPV training and turnover intention [10, 39, 40]. Occupational stress was assessed using the Chinese Nursing Stress Scale (CNSS) developed by Li et al. [41]. This scale includes five dimensions: professional and work‐related stress, workload and time allocation, work environment and resources, patient care and management, and interpersonal relationships, with a total of 35 items. It uses a 4‐point Likert scale scored from 1 (*no stress*) to 4 (*extreme stress*), where higher scores indicate greater stress. Cronbach’s α for this study was 0.949. Burnout was measured using the Chinese Version of the Maslach Burnout Inventory (MBI), revised by Feng et al. [42]. The scale includes three dimensions: emotional exhaustion, depersonalization, and personal accomplishment, with a total of 22 items. It uses a 7‐point Likert scale, ranging from 0 (*never*) to 6 (*occurs daily*). The personal accomplishment dimension is reverse‐scored. Higher total scores indicate higher occupational burnout. According to the MBI diagnostic criteria, the clinical cutoff scores for each dimension are emotional exhaustion ≥ 27, depersonalization ≥ 8, and personal accomplishment ≤ 24. Burnout was defined as meeting the threshold for at least one dimension. In this study, Cronbach’s α was 0.857. Turnover intention was evaluated by the Turnover Intention Questionnaire (TIQ), which was developed by Michael et al. [43] and revised by Li et al. for Chinese populations. The revised scale consists of three dimensions and six items. It uses a 4‐point Likert scale, ranging from frequently to never, scored from 4 to 1. The total score is the sum of the scores for each item, with higher scores indicating stronger turnover intention. In the present study, Cronbach’s α for the scale was 0.826.

Interpersonal relationship variables comprised family support, colleague support, and nurse–child–caregiver communication conflicts [9, 10, 34, 44].

Organizational environment variables included hospital level, form of employment, income, job satisfaction, workload indicators such as work experience, night shift frequency, and daily patient contact time, and adverse event experiences such as medication errors and basic nursing errors [33, 34, 45, 46].

Policy support variables encompassed hospital provision of WPV training and hospital attitudes to WPV [9, 10, 34, 47].

### 2.6. Data Preparation

Data preparation included quality screening, handling of missing data, and processing of class imbalance. Questionnaires were excluded if they were completed in less than 5 min, the minimum time for attentive completion established during pretesting, contained more than 30% missing responses, showed invariant response patterns, defined as the same response option for more than 80% of scale items or at least 10 consecutive identical responses, or contained logical inconsistencies in key items. Subsequently, out‐of‐range or inconsistent values were identified using predefined range and logic checks. Values that could be verified were corrected, whereas those that could not be resolved were coded as missing. After quality‐control screening, the eligible sample was randomly divided into a training set of 414 participants and a test set of 178 participants at a ratio of 7:3. Subsequently, given the very low proportion of missing data (< 1%), single imputation was used in the primary analysis to retain the analyzable sample, with mean imputation for continuous variables and mode imputation for categorical variables. The ratio of nurses with moderate‐to‐high frequency WPV to those without moderate‐to‐high frequency WPV was approximately 1:6, indicating a substantial class imbalance. This imbalance may reduce the model’s ability to detect minority‐class cases. To address this, SMOTE was applied exclusively to the training set as a complementary resampling strategy, while the test set retained its original class distribution to prevent data leakage and allow model performance evaluation under the observed outcome distribution. SMOTE was applied to the training set using three nearest neighbors (*k* = 3). We selected a relatively small *k* value to favor localized interpolation, because smaller neighborhoods constrain synthetic sample generation to observations closer to the original minority‐class instances, whereas broader neighborhoods may increase the risk of interpolation across heterogeneous or overlapping regions and generate noisy synthetic observations [48, 49]. A target minority‐to‐majority class ratio of approximately 1:1 was set, and the oversampling proportion was determined adaptively based on the observed class distribution in the training set. After SMOTE resampling, the training set had an approximately balanced class distribution. Because SMOTE alters the original class distribution, probability estimates and calibration results from the SMOTE‐based model were interpreted cautiously [50].

### 2.7. Analytical Methods

Statistical analyses were performed using SPSS 26.0 and R 4.2.1. Categorical variables were described as frequencies and percentages, and intergroup comparisons were evaluated using chi‐square tests or Fisher’s exact tests. Normally distributed continuous variables were summarized as means and standard deviations and compared using independent‐samples *t* tests, whereas continuous variables with skewed distributions were summarized as medians (Q1, Q3) and compared using Mann–Whitney *U* tests. Variables showing significant associations in the univariate analyses were entered into multivariable logistic regression models. The primary model was fitted using the original training set, whereas a complementary model was fitted using the SMOTE‐resampled training set. Multicollinearity among the variables included in the primary model was assessed using variance inflation factors (VIFs), with a threshold of 5 indicating potential multicollinearity. Nomograms were constructed using coefficients from the primary and SMOTE‐based models, respectively. The primary nomogram was used to estimate the probability of moderate‐to‐high frequency WPV, whereas the SMOTE‐based nomogram served as a complementary comparison to assess the consistency of the effect directions and relative magnitudes of the variable associations after correcting for class imbalance. Furthermore, an interactive web‐based calculator based on the primary model was developed using the Shiny package and deployed via shinyapps.io.

Model performance was evaluated on the training and test sets for the primary model and on the resampled training set and the original test set for the SMOTE‐based model. Model discrimination was assessed using receiver operating characteristic (ROC) curves and AUC. Optimal cutoff values were determined by maximizing the Youden index in the training set and were then applied to the corresponding test set. Sensitivity, specificity, positive predictive value (PPV), negative predictive value (NPV), and accuracy were calculated from the confusion matrices. Calibration was assessed using the Hosmer–Lemeshow test (*p* > 0.05 indicating no significant evidence of lack of fit), and calibration curves were generated using 1000 bootstrap resamples to assess the consistency between predicted probabilities and observed outcomes. Decision curve analysis (DCA) was used to quantify the net benefit across threshold probabilities. Based on the optimal cutoff values, participants were further classified into low‐ and high‐risk groups, and differences in the proportion of moderate‐to‐high frequency WPV between risk groups were compared using chi‐square tests.

Three sensitivity analyses were conducted. First, multiple imputation by chained equations (MICE) was performed on the original training data, generating 20 imputed datasets. The primary logistic regression model was fitted to each dataset, and regression estimates were pooled using Rubin’s rules and compared with the primary analysis. Second, work experience was added to the primary model to assess potential confounding, as longer experience may reflect greater opportunities for exposure to WPV over time. Its contribution was evaluated using a likelihood‐ratio test, changes in the original coefficients and odds ratios (ORs), and model performance in the training and test sets. Discrimination was compared using AUCs and paired DeLong tests, while test‐set calibration and overall performance were assessed using calibration‐in‐the‐large (CITL), calibration slopes, and Brier scores. Third, the robustness of SMOTE to the k‐nearest‐neighbor parameter was examined using *k* = 3, 5, and 7. For each setting, SMOTE was applied only to the training set at a 1:1 class ratio across the same 100 random seeds, and the resulting models were evaluated on the original test set using AUC, Brier score, calibration slope, and coefficient direction. Pairwise differences in AUC between k settings were calculated within matched random seeds, and uncertainty was summarized using 95% empirical percentile intervals. All tests were two‐sided (*α* = 0.05).

### 2.8. Supplementary Contextual Assessment

To provide additional context regarding differences across hospitals, supplementary data were collected from multiple sources. Aggregate numbers of hospital staff and security officers were obtained from the designated administrative departments of each participating hospital. Information on hospital attitudes to WPV and perceived precipitating factors was obtained from corresponding structured items in the nurse questionnaire. Additionally, focus group discussions were conducted with 10 pediatric nursing managers from the participating hospitals. Using a semistructured discussion guide, participants were asked to describe the implementation of entrance‐screening procedures, security staffing and patrol arrangements, and standardized security‐response protocols in their hospitals, with particular attention to challenges and limitations encountered in routine practice. Supplementary contextual data were analyzed separately from the risk stratification model development. Security staffing was expressed as the staff‐to‐security‐officer ratio for each hospital, and grade‐level estimates were obtained by averaging these hospital‐specific ratios. The focus group data were summarized using descriptive content analysis to identify recurring themes in the implementation of WPV prevention practices across hospital levels. Other structured contextual items were summarized by hospital level using frequencies and percentages. These contextual data were neither used for candidate variable selection nor included in the regression models.

### 2.9. Ethical Approval

The study was approved by the Ethics Committee of the Binzhou Medical University (approval number: 2021‐352). Although ethical approval was obtained in 2021, data collection was postponed to February–March 2024 due to restrictions related to COVID‐19. Participant recruitment and data collection were conducted under the approved study protocol.

## 3. Results

### 3.1. Participant Characteristics

A total of 28 invalid questionnaires were excluded, of which 25 showed systematic response patterns, and 3 had extensive missing data. Ultimately, 592 valid questionnaires were included, resulting in a valid response rate of 95.48% (592/620). 56.08% of the participants were aged 30–39 years, 99.49% were female, 76.35% were married, and 65.54% were contract‐based nurses. Additionally, 61.99% worked at least two night shifts per week, and 54.39% spent over 75% of their daily working hours directly interacting with patients. 43.24% reported nurse–child–caregiver communication conflicts, and 14.53% perceived hospital responses as blaming staff. The average score for occupational stress was 72.46 ± 15.20. Only 10.14% reported no occupational burnout, and 4.90% expressed extremely low turnover intention. The prevalence of moderate‐to‐high frequency WPV was 15.46% in the training set and 14.04% in the test set, with no significant difference between the two sets (*p* = 0.659). There were no statistically significant differences in the remaining variables between groups (*p* > 0.05, Table 1).

**TABLE 1:** Baseline characteristics of the training and test sets.

Variable	Description	Total (*n* = 592)	Training set (*n* = 414)	Test set (*n* = 178)	Statistic	*p*
Age (years)	< 30	189 (31.93%)	125 (30.19%)	64 (35.96%)	1.902^b^	0.386
	30–39	332 (56.08%)	238 (57.49%)	94 (52.81%)		
	≥ 40	71 (11.99%)	51 (12.32%)	20 (11.24%)		

Gender	Male	3 (0.51%)	3 (0.72%)	0 (0.00%)	—^d^	0.558
	Female	589 (99.49%)	411 (99.28%)	178 (100.00%)		

Marital status	Unmarried	131 (22.13%)	86 (20.77%)	45 (25.28%)	1.594^d^	0.432
	Married	452 (76.35%)	321 (77.54%)	131 (73.60%)		
	Divorced	9 (1.52%)	7 (1.69%)	2 (1.12%)		

Highest education	Associate degree or below	83 (14.02%)	56 (13.53%)	27 (15.17%)	0.278^b^	0.598
	Bachelor’s degree or above	509 (85.98%)	358 (86.47%)	151 (84.83%)		

Personality type	Introversion	144 (24.32%)	99 (23.91%)	45 (25.28%)	0.199^b^	0.905
	Extroversion	115 (19.43%)	82 (19.81%)	33 (18.54%)		
	Instability	333 (56.25%)	233 (56.28%)	100 (56.18%)		

Self‐perceived health status	Good	289 (48.82%)	202 (48.79%)	87 (48.88%)	0.294^b^	0.864
	Fair	220 (37.16%)	152 (36.71%)	68 (38.20%)		
	Poor	83 (14.02%)	60 (14.49%)	23 (12.92%)		

Willingness to attend WPV training	No	51 (8.61%)	38 (9.18%)	13 (7.30%)	0.556^b^	0.456
	Yes	541 (91.39%)	376 (90.82%)	165 (92.70%)		

Perceived family care	Very high	261 (44.09%)	182 (43.96%)	79 (44.38%)	0.246^d^	0.977
	High	256 (43.24%)	178 (43.00%)	78 (43.82%)		
	Medium	71 (11.99%)	51 (12.32%)	20 (11.24%)		
	Low	4 (0.68%)	3 (0.72%)	1 (0.56%)		

Perceived colleague care	Very high	117 (19.76%)	83 (20.05%)	34 (19.10%)	0.909^d^	0.825
	High	270 (45.61%)	192 (46.38%)	78 (43.82%)		
	Medium	194 (32.77%)	132 (31.88%)	62 (34.83%)		
	Low	11 (1.86%)	7 (1.69%)	4 (2.25%)		

Nurse–child–caregiver communication conflicts	No	336 (56.76%)	243 (58.70%)	93 (52.25%)	2.109^b^	0.146
	Yes	256 (43.24%)	171 (41.30%)	85 (47.75%)		

Hospital level	Class III grade B hospital	147 (24.83%)	101 (24.40%)	46 (25.84%)	0.140^b^	0.709
	Class III grade A hospital	445 (75.17%)	313 (75.60%)	132 (74.16%)		

Form of employment	Permanent staff	157 (26.52%)	114 (27.54%)	43 (24.16%)	2.188^b^	0.335
	Agency staff	47 (7.94%)	36 (8.70%)	11 (6.18%)		
	Contract‐based staff	388 (65.54%)	264 (63.77%)	124 (69.66%)		

Monthly income (yuan)	1000–2999	71 (11.99%)	53 (12.80%)	18 (10.11%)	3.274^b^	0.351
	3000–4999	244 (41.22%)	161 (38.89%)	83 (46.63%)		
	5000–6999	168 (28.38%)	122 (29.47%)	46 (25.84%)		
	≥ 7000	109 (18.41%)	78 (18.84%)	31 (17.42%)		

Job satisfaction	Satisfied	291 (49.16%)	200 (48.31%)	91 (51.12%)	0.454^b^	0.797
	Neutral	243 (41.05%)	172 (41.55%)	71 (39.89%)		
	Dissatisfied	58 (9.80%)	42 (10.14%)	16 (8.99%)		

Night shift frequency (per week)	0	107 (18.07%)	75 (18.12%)	32 (17.98%)	4.931^b^	0.294
	1	118 (19.93%)	78 (18.84%)	40 (22.47%)		
	2	253 (42.74%)	175 (42.27%)	78 (43.82%)		
	3	62 (10.47%)	43 (10.39%)	19 (10.67%)		
	≥ 4	52 (8.78%)	43 (10.39%)	9 (5.06%)		

Patient contact time (daily)	> 75%	322 (54.39%)	225 (54.35%)	97 (54.49%)	0.880^d^	0.644
	30%–75%	255 (43.07%)	180 (43.48%)	75 (42.13%)		
	< 30%	15 (2.53%)	9 (2.17%)	6 (3.37%)		

Work experience (year)	< 5	221 (37.33%)	156 (37.68%)	65 (36.52%)	0.495^b^	0.781
	5–10	190 (32.09%)	135 (32.61%)	55 (30.90%)		
	> 10	181 (30.57%)	123 (29.71%)	58 (32.58%)		

Medication errors	No	312 (52.70%)	213 (51.45%)	99 (55.62%)	0.868^b^	0.352
	Yes	280 (47.30%)	201 (48.55%)	79 (44.38%)		

Basic nursing errors	No	429 (72.47%)	300 (72.46%)	129 (72.47%)	< 0.001^b^	0.998
	Yes	163 (27.53%)	114 (27.54%)	49 (27.53%)		

Hospital provision of WPV training	No	309 (52.20%)	220 (53.14%)	89 (50.00%)	0.492^b^	0.483
	Yes	283 (47.80%)	194 (46.86%)	89 (50.00%)		

Hospital attitudes to WPV	Proactive protection of employee rights	106 (17.91%)	77 (18.60%)	29 (16.29%)	2.051^b^	0.562
	Delayed responses	352 (59.46%)	240 (57.97%)	112 (62.92%)		
	Tolerance of violence	48 (8.11%)	37 (8.94%)	11 (6.18%)		
	Blaming employees	86 (14.53%)	60 (14.49%)	26 (14.61%)		

Occupational stress		72.46 ± 15.20	72.53 ± 15.13	72.29 ± 15.40	0.174^c^	0.862

Burnout	No	60 (10.14%)	41 (9.90%)	19 (10.67%)	0.081^b^	0.776
	Yes	532 (89.86%)	373 (90.10%)	159 (89.33%)		

Turnover intention		15.00 (12.00,17.00%)	15.00 (12.00,17.00)	15.00 (11.00,17.00)	−0.033^a^	0.973

Moderate‐to‐high frequency WPV	No	503 (84.97%)	350 (84.54%)	153 (85.96%)	0.195^b^	0.659
	Yes	89 (15.03%)	64 (15.46%)	25 (14.04%)		

*Note:* a = *Z*, b = *χ* ^2^, c = *t*, d = Fisher.

### 3.2. Univariate Analysis

Table 2 presents the results of the univariate analysis. Significant differences were observed in self‐perceived health status, nurse–child–caregiver communication conflicts, hospital level, job satisfaction, hospital attitudes to WPV, occupational stress, and burnout between nurses with and without moderate‐to‐high frequency WPV (*p* < 0.05).

**TABLE 2:** Univariate analysis of candidate variables associated with moderate‐to‐high frequency WPV in the training set.

Variable	Description	Total (*n* = 414)	Moderate‐to‐high frequency WPV		Statistic	*p*
			Yes (*n* = 64)	No (*n* = 350)		
Age (years)	< 30	125 (30.19%)	15 (23.44%)	110 (31.43%)	5.091^b^	0.078
	30–39	238 (57.49%)	36 (56.25%)	202 (57.71%)		
	≥ 40	51 (12.32%)	13 (20.31%)	38 (10.86%)		

Gender	Male	3 (0.72%)	1 (1.56%)	2 (0.57%)	—^d^	0.397
	Female	411 (99.28%)	63 (98.44%)	348 (99.43%)		

Marital status	Unmarried	86 (20.77%)	8 (12.50%)	78 (22.29%)	3.284^d^	0.171
	Married	321 (77.54%)	55 (85.94%)	266 (76.00%)		
	Divorced	7 (1.69%)	1 (1.56%)	6 (1.71%)		

Highest education	Associate degree or below	56 (13.53%)	5 (7.81%)	51 (14.57%)	2.113^b^	0.146
	Bachelor’s degree or above	358 (86.47%)	59 (92.19%)	299 (85.43%)		

Personality type	Introversion	99 (23.91%)	16 (25.00%)	83 (23.71%)	0.841^b^	0.657
	Extroversion	82 (19.81%)	15 (23.44%)	67 (19.14%)		
	Instability	233 (56.28%)	33 (51.56%)	200 (57.14%)		

Self‐perceived health status	Good	202 (48.79%)	19 (29.69%)	183 (52.29%)	11.971^b^	**0.003**
	Fair	152 (36.71%)	30 (46.88%)	122 (34.86%)		
	Poor	60 (14.49%)	15 (23.44%)	45 (12.86%)		

Willingness to attend WPV training	No	38 (9.18%)	8 (12.50%)	30 (8.57%)	1.002^b^	0.317
	Yes	376 (90.82%)	56 (87.50%)	320 (91.43%)		

Perceived family care	Very high	182 (43.96%)	22 (34.38%)	160 (45.71%)	7.182^d^	0.055
	High	178 (43.00%)	32 (50.00%)	146 (41.71%)		
	Medium	51 (12.32%)	8 (12.50%)	43 (12.29%)		
	Low	3 (0.72%)	2 (3.13%)	1 (0.29%)		

Perceived colleague care	Very high	83 (20.05%)	11 (17.19%)	72 (20.57%)	2.285^d^	0.473
	High	192 (46.38%)	33 (51.56%)	159 (45.43%)		
	Medium	132 (31.88%)	18 (28.13%)	114 (32.57%)		
	Low	7 (1.69%)	2 (3.13%)	5 (1.43%)		

Nurse–child–caregiver communication conflicts	No	243 (58.70%)	21 (32.81%)	222 (63.43%)	20.919^b^	**< 0.001**
	Yes	171 (41.30%)	43 (67.19%)	128 (36.57%)		

Hospital level	Class III grade B hospital	101 (24.40%)	23 (35.94%)	78 (22.29%)	5.467^b^	**0.019**
	Class III grade A hospital	313 (75.60%)	41 (64.06%)	272 (77.71%)		

Form of employment	Permanent staff	114 (27.54%)	22 (34.38%)	92 (26.29%)	1.997^b^	0.368
	Agency staff	36 (8.70%)	6 (9.38%)	30 (8.57%)		
	Contract‐based staff	264 (63.77%)	36 (56.25%)	228 (65.14%)		

Monthly income (yuan)	1000–2999	53 (12.80%)	4 (6.25%)	49 (14.00%)	4.981^b^	0.173
	3000–4999	161 (38.89%)	25 (39.06%)	136 (38.86%)		
	5000–6999	122 (29.47%)	18 (28.13%)	104 (29.71%)		
	≥ 7000	78 (18.84%)	17 (26.56%)	61 (17.43%)		

Job satisfaction	Satisfied	200 (48.31%)	17 (26.56%)	183 (52.29%)	40.657^b^	**< 0.001**
	Neutral	172 (41.55%)	27 (42.19%)	145 (41.43%)		
	Dissatisfied	42 (10.14%)	20 (31.25%)	22 (6.29%)		

Night shift frequency (per week)	0	75 (18.12%)	15 (23.44%)	60 (17.14%)	1.873^b^	0.759
	1	78 (18.84%)	12 (18.75%)	66 (18.86%)		
	2	175 (42.27%)	25 (39.06%)	150 (42.86%)		
	3	43 (10.39%)	5 (7.81%)	38 (10.86%)		
	≥ 4	43 (10.39%)	7 (10.94%)	36 (10.29%)		

Patient contact time (daily)	> 75%	225 (54.35%)	31 (48.44%)	194 (55.43%)	1.569^d^	0.443
	30%–75%	180 (43.48%)	31 (48.44%)	149 (42.57%)		
	< 30%	9 (2.17%)	2 (3.13%)	7 (2.00%)		

Work experience (year)	< 5	156 (37.68%)	21 (32.81%)	135 (38.57%)	2.221^b^	0.329
	5–10	135 (32.61%)	19 (29.69%)	116 (33.14%)		
	> 10	123 (29.71%)	24 (37.50%)	99 (28.29%)		

Medication errors	No	213 (51.45%)	26 (40.63%)	187 (53.43%)	3.551^b^	0.060
	Yes	201 (48.55%)	38 (59.38%)	163 (46.57%)		

Basic nursing errors	No	300 (72.46%)	47 (73.44%)	253 (72.29%)	0.036^b^	0.850
	Yes	114 (27.54%)	17 (26.56%)	97 (27.71%)		

Hospital provision of WPV training	No	220 (53.14%)	40 (62.50%)	180 (51.43%)	2.663^b^	0.103
	Yes	194 (46.86%)	24 (37.50%)	170 (48.57%)		

Hospital attitudes to WPV	Proactive protection of employee rights	77 (18.60%)	5 (7.81%)	72 (20.57%)	19.373^b^	**< 0.001**
	Delayed responses	240 (57.97%)	32 (50.00%)	208 (59.43%)		
	Tolerance of violence	37 (8.94%)	13 (20.31%)	24 (6.86%)		
	Blaming employees	60 (14.49%)	14 (21.88%)	46 (13.14%)		

Occupational stress		72.53 ± 15.13	81.77 ± 12.39	70.84 ± 14.99	−5.496^c^	**< 0.001**

Burnout	No	41 (9.90%)	1 (1.56%)	40 (11.43%)	5.903^b^	**0.015**
	Yes	373 (90.10%)	63 (98.44%)	310 (88.57%)		

Turnover intention		15.00 (12.00,17.00)	16.00 (13.00,17.75)	15.00 (11.00,17.00)	−1.592^a^	0.111

*Note:* a = *Z*, b = *χ* ^2^, c = *t*, d = Fisher. Bold values indicate statistical significance (*p* < 0.05).

### 3.3. Development of the Primary Multivariable Logistic Regression Model

In the original training set, multivariable logistic regression identified five variables independently associated with moderate‐to‐high frequency WPV: hospital level, nurse–child–caregiver communication conflicts, tolerance of violence, occupational stress, and burnout (Table 3). Multicollinearity diagnostics showed that VIFs for all variables ranged from 1.003 to 1.127, indicating no evidence of substantial multicollinearity.

**TABLE 3:** Multivariable logistic regression analysis of factors associated with moderate‐to‐high frequency WPV in the original training set.

Factors	*β*	SE	OR (95% CI)	*p*
(Constant)	−7.434	1.456	0.001 (< 0.001–0.010)	**< 0.001**
Self‐perceived health status				
Good			1 (Ref)	
Fair	0.552	0.356	1.737 (0.865–3.487)	0.120
Poor	0.667	0.436	1.948 (0.828–4.581)	0.126
Hospital level				
Class III grade B hospital			1 (Ref)	
Class III grade A hospital	−0.667	0.323	0.513 (0.273–0.967)	**0.039**
Nurse–child–caregiver communication conflicts				
No			1 (Ref)	
Yes	1.144	0.312	3.140 (1.705–5.786)	**< 0.001**
Hospital attitudes to WPV				
Proactive protection of employee rights			1 (Ref)	
Delayed responses	0.649	0.524	1.914 (0.685–5.346)	0.215
Tolerance of violence	1.331	0.629	3.784 (1.102–12.992)	**0.034**
Blaming employees	0.686	0.606	1.985 (0.606–6.506)	0.258
Occupational stress	0.034	0.012	1.034 (1. 010–1.059)	**0.005**
Burnout				
No			1 (Ref)	
Yes	2.142	1.039	8.513 (1.111–65.210)	**0.039**

*Note:* Bold values indicate statistical significance (*p* < 0.05).

### 3.4. Development of the Complementary Model Using SMOTE‐Resampled Training Data

To further assess the potential influence of class imbalance, the multivariable logistic regression analysis was additionally performed in the SMOTE‐resampled training set. In the SMOTE‐based dataset, fair health status, poor health status, hospital level, nurse–child–caregiver communication conflicts, delayed hospital attitudes to WPV, tolerance of violence, blaming employees, occupational stress, and burnout were independently associated with moderate‐to‐high frequency WPV. Detailed results are presented in Table 4.

**TABLE 4:** Multivariable logistic regression analysis of factors associated with moderate‐to‐high frequency WPV in the SMOTE‐resampled training set.

Factors	*β*	SE	OR (95% CI)	*p*
(Constant)	−7.349	0.911	0.001 (< 0.001–0.004)	**< 0.001**
Self‐perceived health status				
Good			1 (Ref)	
Fair	0.525	0.202	1.691 (1.137–2.515)	**0.009**
Poor	0.546	0.264	1.727 (1.029–2.897)	**0.039**
Hospital level				
Class III grade B hospital			1 (Ref)	
Class III grade A hospital	−0.477	0.197	0.621 (0.422–0.913)	**0.015**
Nurse–child–caregiver communication conflicts				
No			1 (Ref)	
Yes	1.300	0.182	3.669 (2.570–5.238)	**< 0.001**
Hospital attitudes to WPV				
Proactive protection of employee rights			1 (Ref)	
Delayed response	1.001	0.296	2.722 (1.523–4.865)	**0.001**
Tolerance of violence	1.831	0.385	6.241 (2.936–13.268)	**< 0.001**
Blaming employees	0.831	0.353	2.296 (1.149–4.587)	**0.019**
Occupational stress	0.040	0.008	1.041 (1.025–1.057)	**< 0.001**
Burnout				
No			1 (Ref)	
Yes	2.830	0.629	16.938 (4.934–58.147)	**< 0.001**

*Note:* Bold values indicate statistical significance (*p* < 0.05).

### 3.5. Construction of Nomograms

**FIGURE 1:**
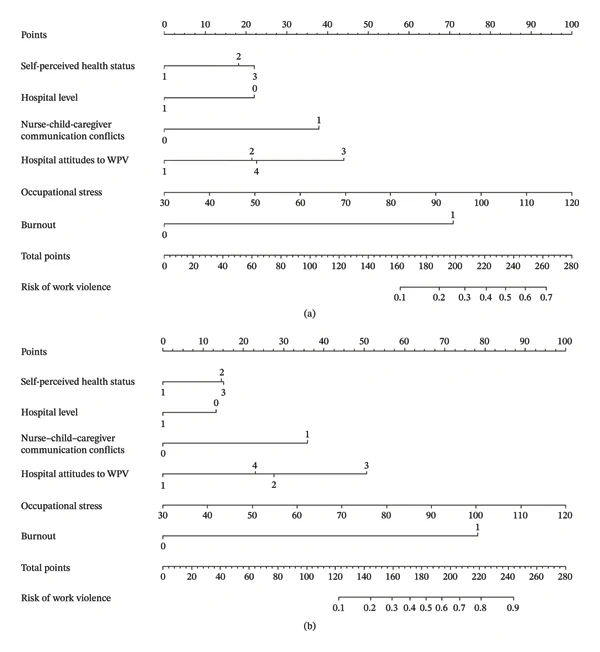
Nomograms for estimating the probability of moderate‐to‐high frequency WPV among pediatric nurses. Note: (a) Nomogram using the original training set; (b) nomogram using the SMOTE‐resampled training set.

Nomograms were developed from the primary and SMOTE‐based models to estimate the probability of moderate‐to‐high frequency WPV. Total points were calculated by summing the scores assigned to each variable. For illustration, in the nomogram shown in Figure 1a, a pediatric nurse working in a Class III Grade B hospital (22 points), with good health status (0 points), nurse–child–caregiver communication conflicts (38 points), delayed hospital attitudes to WPV (22 points), an occupational stress score of 80 (55 points), and burnout (71 points), would have a total score of 208 points, corresponding to an estimated probability of 31%. To improve usability, an interactive web‐based calculator based on the primary model was developed using Shiny and is available at https://moderate-to-high-frequency-wpv.shinyapps.io/WpvRiskTool-1/. After entering the relevant variables, the calculator provides real‐time individualized probability estimates and risk stratification results.

### 3.6. Model Performance

**FIGURE 2:**
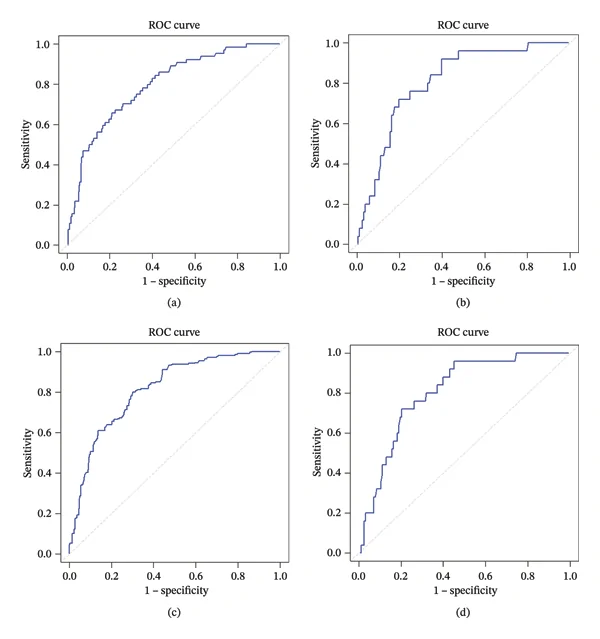
ROC curves of the nomogram‐based models in the training and test sets. Note: (a) Primary model in the training set; (b) primary model in the test set; (c) SMOTE‐based model in the SMOTE‐resampled training set; and (d) SMOTE‐based model in the original test set.

**FIGURE 3:**
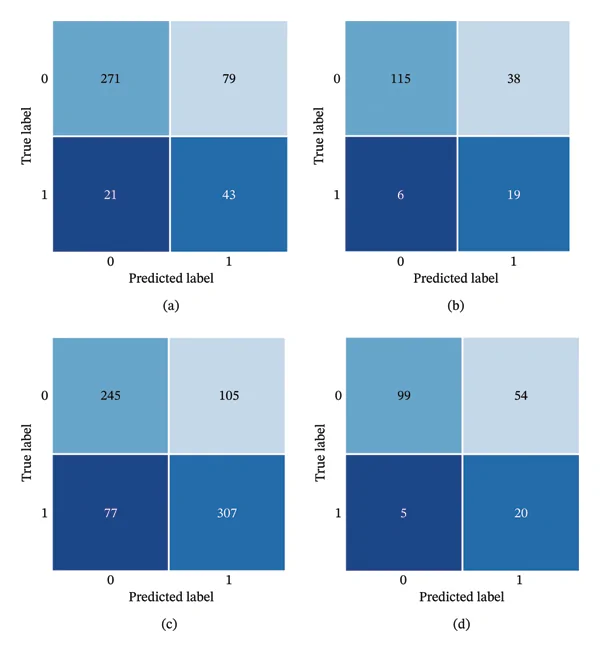
Confusion matrices of the nomogram‐based models in the training and test sets. Note: (a) Primary model in the training set; (b) primary model in the test set; (c) SMOTE‐based model in the SMOTE‐resampled training set; and (d) SMOTE‐based model in the original test set.

**FIGURE 4:**
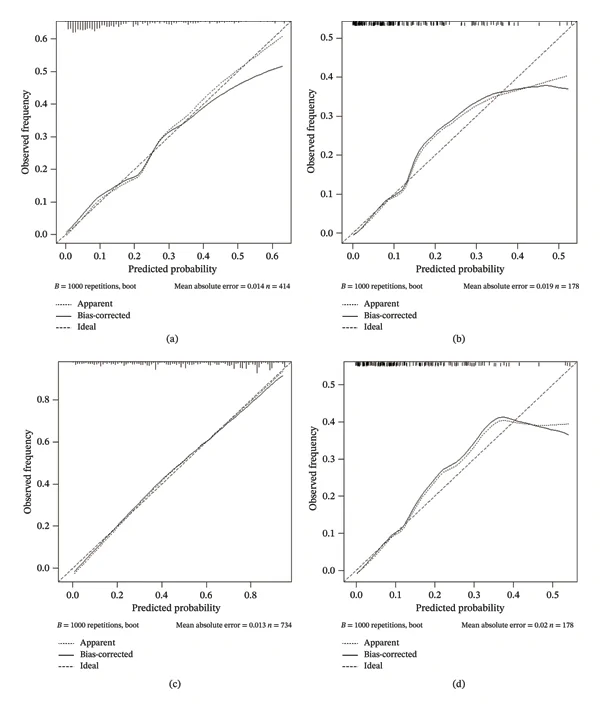
Calibration curves of the nomogram‐based models in the training and test sets. Note: (a) Primary model in the training set; (b) primary model in the test set; (c) SMOTE‐based model in the SMOTE‐resampled training set; and (d) SMOTE‐based model in the original test set.

**FIGURE 5:**
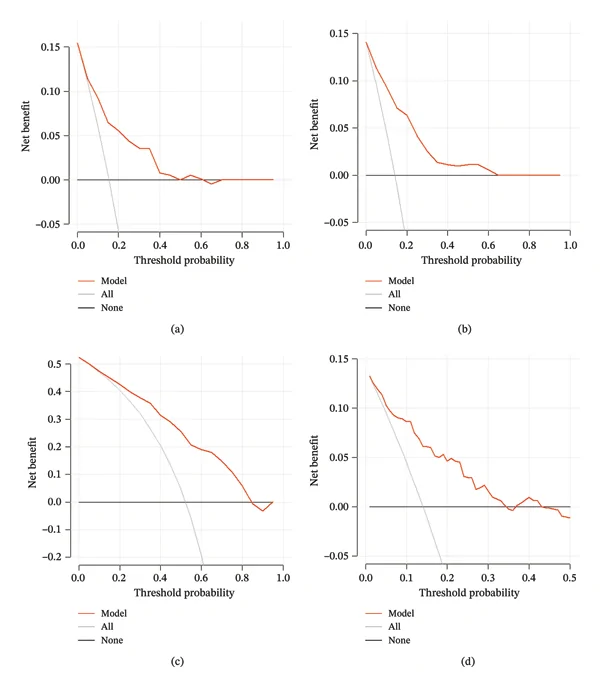
DCA curves of the nomogram‐based models in the training and test sets. Note: (a) Primary model in the training set; (b) primary model in the test set; (c) SMOTE‐based model in the SMOTE‐resampled training set; and (d) SMOTE‐based model in the original test set.

In the primary model, the AUC values were 0.794 (95% confidence interval [CI]: 0.736–0.852) in the training set and 0.808 (95% CI: 0.726–0.890) in the test set (Figure 2). The optimal cutoff was 0.195 (187 points), yielding a sensitivity of 0.672 and a specificity of 0.774 in the training set. In the test set, the sensitivity and specificity were 0.760 and 0.752, respectively. Detailed performance metrics and confusion matrices are shown in Table 5 and Figure 3. Calibration was acceptable in both datasets, as indicated by the Hosmer–Lemeshow test (training set: *χ* ^2^ = 3.106, *p* = 0.928; test set: *χ* ^2^ = 6.150, *p* = 0.630), which was consistent with the calibration curves shown in Figure 4. DCA showed positive net benefit across threshold probabilities of 3%–50% in the training set and 2%–65% in the test set (Figure 5).

**TABLE 5:** Performance of the primary and SMOTE‐based nomogram models in the training and test sets.

Models	Sensitivity (95% CI)	Specificity (95% CI)	PPV (95% CI)	NPV (95% CI)	Accuracy (95% CI)
Primary model (training set)	0.672 (0.576–0.935)	0.774 (0.541–0.908)	0.352 (0.248–0.560)	0.928 (0.912–0.981)	0.759 (0.714–0.799)
Primary model (test set)	0.760 (0.571–0.909)	0.752 (0.682–0.814)	0.333 (0.211–0.453)	0.950 (0.907–0.983)	0.753 (0.683–0.814)
SMOTE‐based model (SMOTE‐resampled training set)	0.799 (0.762–0.841)	0.700 (0.648–0.750)	0.745 (0.703–0.788)	0.761 (0.716–0.805)	0.752 (0.719–0.783)
SMOTE‐based model (original test set)	0.800 (0.632–0.952)	0.647 (0.576–0.719)	0.270 (0.171–0.368)	0.952 (0.907–0.990)	0.669 (0.594–0.737)

In the SMOTE‐based model, the AUC values were 0.814 (95% CI: 0.783–0.845) in the resampled training set and 0.805 (95% CI: 0.725–0.885) in the original test set (Figure 2). The optimal cutoff was 0.485 (181 points on the nomogram). Sensitivity and specificity were 0.799 and 0.700 in the training set, and 0.800 and 0.647 in the test set. The Hosmer–Lemeshow test indicated good calibration in the training set (*χ* ^2^ = 11.128, *p* = 0.195) but poor calibration in the test set (*χ* ^2^ = 95.134, *p* < 0.001). In the test set, the calibration curve indicated reasonable agreement at low‐to‐moderate predicted probabilities, whereas overestimation was observed at higher probabilities (Figure 4). DCA showed positive net benefit across threshold probabilities of 5%–85% in the training set and 2%–34% in the test set (Figure 5).

### 3.7. Risk Stratification Based on the Nomograms

Using the cutoff of 0.195, the primary model classified nurses into low‐ and high‐risk groups. The prevalence of moderate‐to‐high frequency WPV was higher in the high‐risk group than in the low‐risk group in both the training set (35.25% vs. 7.19%, *χ* ^2^ = 49.695, *p* < 0.001) and the test set (33.33% vs. 4.96%, *χ* ^2^ = 23.544, *p* < 0.001). Using the cutoff of 0.485, the SMOTE‐based model yielded corresponding prevalence values of 74.51% and 23.91% in the training set (*χ* ^2^ = 183.49, *p* < 0.001). When applied to the original test set, the prevalence was 27.03% in the high‐risk group and 4.81% in the low‐risk group (*χ* ^2^ = 15.889, *p*  <  0.001), indicating effective stratification under the original class distribution.

### 3.8. Sensitivity Analyses

The pooled regression estimates from the MICE analysis were consistent in direction and similar in magnitude to those from the primary analysis (Supporting File 2). All variables that were statistically significant in the primary analysis remained significant after multiple imputation. These findings suggested that the results were not materially affected by the method used to handle missing data.

Adding work experience as a categorical variable did not significantly improve model fit (likelihood‐ratio test, *p* = 0.369). Neither 5–10 years nor > 10 years was independently associated with WPV (*p* = 0.688 and *p* = 0.172, respectively), with original coefficients remaining stable (Supporting File 3). In the training set, AUCs were similar (0.794 vs. 0.795; DeLong *p* = 0.896). In the test set, the adjusted model had a lower AUC (0.785 vs. 0.808; DeLong *p* = 0.017), though calibration remained comparable (CITL: −0.158 vs. −0.164; slope: 1.105 vs. 1.004; Brier: 0.104 vs. 0.106). Given the lack of improvement, the parsimonious primary model was retained.

Sensitivity analysis demonstrated stable model performance across the tested SMOTE *k* values. Median AUCs were 0.805, 0.801, and 0.802 for *k* = 3, 5, and 7, respectively. The 95% empirical percentile intervals for pairwise AUC differences included zero, providing no evidence of systematic differences in discrimination. Median Brier scores ranged from 0.199 to 0.204 across the tested settings, and median calibration slopes ranged from 0.933 to 0.992. The direction of variable coefficients remained consistent across all three k values. Overall, the model was insensitive to the tested SMOTE *k* values.

### 3.9. Supplementary Contextual Results

To characterize the institutional context of the participating hospitals, supplementary administrative and nurse‐reported data were descriptively examined across the 10 study hospitals, including 7 Class III Grade A and 3 Class III Grade B hospitals. The mean staff‐to‐security‐officer ratio was higher in Class III Grade B hospitals than in Class III Grade A hospitals (17.80 vs. 14.50). Additional structured nurse‐reported data showed higher proportions of employee‐blaming responses (16.33% [24/147] vs. 13.93% [62/445]) and treatment dissatisfaction as a perceived WPV precipitant (19.73% [29/147] vs. 13.71% [61/445]) but a lower proportion of proactive protection of employee rights (12.93% [19/147] vs. 19.55% [87/445]) in Class III Grade B hospitals. Descriptive content analysis identified several recurring themes across hospital levels. Managers from Class III Grade B hospitals described limitations in the implementation of outpatient entrance‐screening measures, access control at pediatric ward entrances, security staffing and patrol coverage, and the standardization of emergency response procedures. Specific concerns included infrequent use of screening equipment in some Class III Grade B hospitals, with greater reliance on visual inspection of personal belongings by security staff; open ward entrances without dedicated personnel to monitor or register entry and exit; security personnel being shared across multiple departments; and unclear operational steps and staff responsibilities during emergency responses. For example, one Grade B manager noted, “There is no one permanently stationed at the pediatric ward entrance. One security guard covers three departments, so if a family member becomes agitated, it may take some time for someone to arrive” (M3). In contrast, managers from Class III Grade A hospitals described variability in the implementation of entrance‐screening procedures across different times of day, as well as insufficient scenario‐specific training and drills. One Grade A manager stated, “We have screening equipment and policies, but at night, when there are fewer people on‐site, some screening procedures are implemented less consistently” (M5).

## 4. Discussion

WPV against nurses represents a major global public health challenge, particularly among high‐risk groups such as pediatric nurses. Guided by the SEM, this cross‐sectional study developed and validated nomogram‐based models for concurrent risk stratification of moderate‐to‐high frequency WPV among Chinese pediatric nurses. In the primary model, hospital level, nurse–child–caregiver communication conflicts, tolerance of violence, occupational stress, and burnout were independently associated with moderate‐to‐high frequency WPV. A complementary SMOTE‐based analysis additionally identified fair health status, poor health status, delayed hospital attitudes to WPV, and blaming employees. Given the cross‐sectional design, these associated factors should not be interpreted as definitive antecedents of WPV. In particular, occupational stress, burnout, and self‐perceived health may reflect consequences of repeated WPV exposure or mutually reinforcing relationships. Overall, the nomogram may provide a practical reference for identifying nurses with a higher probability of recent moderate‐to‐high frequency WPV exposure and for informing targeted prevention and management strategies.

Self‐perceived health status was independently associated with moderate‐to‐high frequency WPV only in the SMOTE‐based analysis. This finding is partly consistent with Li et al. [51], who reported that poor general well‐being was associated with WPV among nurses. Health problems such as chronic pain, fatigue, or insufficient sleep may be related to reduced emotional regulation and attention, which family members may perceive as insufficient patience or reduced professionalism [52, 53]. Additionally, poorer self‐perceived health may also be accompanied by lower energy, slower responses, or reduced eye contact. According to the target congruence theory, perpetrators may interpret such signs as indicators of vulnerability in conflict situations, which could help account for the elevated WPV risk among nurses with poorer health [17, 54]. However, this finding should be interpreted cautiously, as the association was observed only in the SMOTE‐based analysis and may be influenced by resampling. Therefore, this result should be regarded as exploratory and interpreted in conjunction with other factors. In practice, self‐perceived health status can serve as a supplementary indicator to help managers identify nurses who may need additional support. For nurses reporting poorer perceived health, managers may pay closer attention during routine communication, safety debriefing, and review of WPV‐related incidents.

The present study found that nurses working in Class III Grade A hospitals had a significantly lower likelihood of moderate‐to‐high frequency WPV than those working in Class III Grade B hospitals. This finding is similar to Tian et al. [55], but differs from Xiao et al. [56]. These discrepancies may reflect differences in institutional resources [57] and care contexts. Exploratory contextual analyses indicated lower security staffing density in Class III Grade B hospitals, while focus group discussions with nursing managers suggested greater constraints in access control, security coverage, and standardized emergency‐response procedures. By contrast, challenges in Class III Grade A hospitals appeared to relate more to the consistent implementation of existing preventive measures and the adequacy of scenario‐based training. Structured nurse‐reported data further suggested less supportive organizational responses to WPV and a higher proportion of nurses identifying treatment dissatisfaction as a perceived precipitating factor in Class III Grade B hospitals. Together, these findings suggest that differences in security resources, response capacity, and organizational support may be associated with the higher WPV risk observed in Class III Grade B hospitals. This may be particularly relevant in pediatric settings, where the strong emotional involvement of parents and other family members may heighten tension in clinical interactions when expectations regarding care are unmet. When combined with limited security coverage or response capacity, such tensions may be more difficult to contain before they escalate into WPV [10]. Accordingly, prevention strategies in hospitals with relatively limited resources could include ensuring timely access to security and managerial support, strengthening the consistent implementation of entrance‐screening and access‐control measures, standardizing emergency response and WPV‐reporting procedures, providing scenario‐based training, and ensuring nonpunitive postincident support for affected nurses. Targeted institutional or regional resource support may also help reduce disparities in the capacity to prevent WPV across hospital levels [47, 58]. Because these supplementary analyses were exploratory and based on a limited number of hospitals, the role of these institutional factors should be examined further in larger multilevel studies.

Pediatric nurses who had experienced nurse–child–caregiver communication conflicts were more likely to experience moderate‐to‐high frequency WPV. Aljohani et al. [59] found that ineffective communication may contribute to WPV, which is consistent with the present findings. Several explanations may account for this association. First, families’ anxiety arising from uncertainty about disease prognosis, pain control, and financial burden may be exacerbated by unclear or defensive communication [60]. Second, ineffective communication may lead to misunderstanding of medical information among patients and families, fostering distrust and hostility [8]. These communication challenges may be particularly salient in pediatric settings. Unlike adult care, pediatric nursing often involves triadic communication among nurses, children, and caregivers [61]. Nurses must explain care procedures to caregivers while simultaneously using developmentally appropriate approaches to communicate with children, such as nonverbal soothing for infants, simple language and distraction for preschool children, and more detailed explanations for school‐aged children and adolescents. In addition, caregivers in pediatric settings often have heightened emotional involvement and stronger expectations regarding comfort, safety, and timely responses, particularly during procedures such as venipuncture or infusion. Therefore, pediatric nursing training should emphasize family‐centered communication, age‐specific communication strategies, structured preprocedural communication, and conflict de‐escalation skills [62]. Feasible approaches include standardized communication scripts [63], role‐play training [64], structured preprocedural explanations [65], and senior‐nurse support during high‐risk procedures [66]. Where resources permit, newer approaches such as VR‐based training may be explored as optional tools for simulating complex communication scenarios [67, 68].

Organizational attitudes to WPV were also important. In the primary model, tolerance of violence was independently associated with moderate‐to‐high frequency WPV. In the SMOTE‐based model, delayed handling and blaming employees also became significant. These results, which are similar to Thomas et al. [69], suggest that perceived institutional responses to WPV may be closely related to nurses’ repeated exposure to violence. Management tolerance may normalize violence, reduce nurses’ willingness to report incidents [17], and weaken institutional deterrence. Similarly, delayed responses or blaming employees may be perceived by perpetrators as an indication that they will not be held accountable [17], which may be associated with the escalation of violence. Despite increasing policy emphasis on WPV prevention and institutional response capacity [70], only 17.91% of participants reported active protection of nurses’ rights, whereas approximately 8.11% and 14.53% reported tolerance of violence and blaming employees, respectively. These findings may indicate a perceived gap between policy expectations and institutional support experienced by nurses in practice. To address this gap, hospitals may need to strengthen zero‐tolerance policies, establish nonpunitive reporting channels, implement timely response procedures, provide postincident support, and reinforce managerial accountability [71]. Additional measures, such as scenario‐based drills or emergency alarm systems, may be considered according to institutional resources and local risk levels [72–74].

This study found that occupational stress was independently associated with moderate‐to‐high frequency WPV. This finding is consistent with O’Brien et al. [75], who suggested that high workload, staffing shortages, and stressful clinical environments are associated with WPV among nurses. From the perspective of the transactional model of stress and coping [76], stress reflects a dynamic person‐environment process in which nurses appraise environmental demands in relation to their available coping resources. In pediatric care, nurses must manage clinical tasks while simultaneously responding to children’s care needs and caregivers’ information needs, anxiety, and expectations for prompt attention [77]. When perceived work demands exceed available coping resources, occupational stress may affect service delivery by limiting nurses’ time, responsiveness, and ability to provide timely care. Such delays or insufficient explanations may be perceived by caregivers as unmet care expectations, which may be associated with dissatisfaction, tension, or conflict [75]. These findings suggest that occupational stress should be considered when developing WPV prevention strategies.

Nurses with burnout had a higher likelihood of moderate‐to‐high frequency WPV than those without burnout, which is consistent with Nombera‐Aznaran et al. [38]. Burnout is commonly characterized by emotional exhaustion, depersonalization, and reduced professional efficacy [78]. These dimensions may help explain the observed association between burnout and WPV. First, emotional exhaustion and reduced professional efficacy may be associated with greater difficulty maintaining empathic communication and de‐escalating conflicts in nurse‐family encounters [79, 80]. Second, depersonalization may be reflected in more detached or less engaged interpersonal responses, which may be perceived negatively by family members and may be related to lower trust or greater tension [81]. The high prevalence of burnout in this study (89.86%) indicates a substantial occupational health burden among pediatric nurses. This proportion appears higher than that reported by Shah et al. [82] and Quesada‐Puga et al. [83], which may reflect differences in study populations, clinical settings, and approaches to measuring burnout. Therefore, WPV prevention should be integrated with occupational health support. For lower risk nurses, universal support may include workload review, reasonable scheduling, and access to stress‐management resources [84]. For higher risk nurses, targeted measures may be considered, including psychological counseling, evidence‐based stress‐management programs, and, when needed, referral to occupational health services [85, 86]. Additionally, unlike previous studies [39, 87], the univariate analysis in the present study showed no statistically significant association between WPV and turnover intention among pediatric nurses. This finding may partly reflect the healthy worker survivor effect, as only currently employed nurses were included.

From a practical perspective, the nomogram‐based risk stratification models translated multidimensional associated factors into individualized risk scores, which may improve the interpretability of risk stratification compared with single‐indicator assessments. The primary model showed stable discrimination, with AUC values of 0.794 and 0.808 in the training and test sets, respectively, and acceptable calibration in both datasets. Therefore, it was considered the primary model for risk stratification and probability estimation under the observed class distribution. The SMOTE‐based model showed comparable discrimination and higher sensitivity, which may be useful for reducing missed identification of potentially high‐risk nurses, particularly when the screening goal prioritizes sensitivity over precise probability estimation. However, it also showed lower specificity and poor calibration in the original test set, with overestimation at higher predicted probabilities. This is consistent with methodological evidence that imbalance correction methods, including SMOTE, may overestimate minority‐class probabilities and produce poorly calibrated risk estimates [50]. Therefore, the SMOTE‐based model should be interpreted as a complementary model. Sensitivity analyses further indicated that the study findings were robust to alternative missing‐data handling, the inclusion of work experience, and the choice of SMOTE *k* values. Using the primary cutoff of 0.195, corresponding to 187 nomogram points, the primary model stratified nurses into low‐ and high‐risk groups with significantly different observed prevalence of moderate‐to‐high frequency WPV in both the training and test sets. DCA showed that both models provided net benefit across a common threshold range of 5%–34% in both the training and test sets. This range may therefore serve as a preliminary reference interval for WPV risk stratification. Within this interval, the primary model cutoff of 19.5% may be considered a practical operating threshold, as it was derived by maximizing the Youden index and fell within the net‐benefit ranges in both the training and test sets. Nevertheless, this threshold should not be regarded as universally applicable and may require adaptation according to local resources, and implementation priorities. Nurses classified as high risk may be considered for targeted managerial support, such as communication training, timely reporting channels, psychological support, stress management, and burnout reduction programs. At the organizational level, the findings, particularly those related to institutional responses and tolerance of violence, highlight the need for hospitals to strengthen zero‐tolerance policies, rapid response procedures, nonpunitive support systems, and accountability mechanisms for WPV prevention. Finally, the Shiny calculator developed in this study may facilitate individualized risk estimation and risk stratification, thereby supporting managerial decision‐making.

## 5. Limitations

This study has several limitations. First, the cross‐sectional design limits causal and temporal inference. Although the nomograms can stratify nurses according to their estimated probability of moderate‐to‐high frequency WPV during the preceding 12 months, they cannot determine whether the included factors preceded WPV exposure. In particular, occupational stress, burnout, and self‐rated health may be both consequences and correlates of repeated exposure. Therefore, the models should be interpreted as concurrent risk‐stratification tools, rather than prediction models for future WPV occurrence. Prospective cohort studies with repeated measurements are needed to clarify temporal relationships. Second, although the SMOTE‐based model was evaluated in the original test set, its findings should be interpreted with caution. The larger effect estimates observed for some variables, particularly burnout, may reflect resampling‐induced amplification, especially for variables conceptually related to the outcome. Because SMOTE generates synthetic observations from a limited number of minority‐class cases and changes the original class distribution, it may increase overfitting risk, affect coefficient estimation, and yield estimated probabilities that do not fully align with the original population prevalence. These issues may limit generalizability under real‐world conditions. Therefore, the SMOTE‐based model should be regarded as a complementary analysis. Third, although this was a multicenter study, the model was developed and internally validated within the same study population. Its transportability to other regions and hospital types remains uncertain. As a next step, we plan to externally validate the nomogram in an independent sample of pediatric nurses from Class III Grade A and Grade B hospitals in at least two provinces outside Shandong. A preliminary precision‐based calculation indicated that approximately 1400 participants would be required, before adjustment for hospital‐level clustering and incomplete data. The original model equation and the cutoff will be applied unchanged, and discrimination, calibration, and net benefit will be evaluated. The SMOTE‐based model will be assessed as a complementary analysis, with particular attention to calibration under the original outcome distribution. Where feasible, performance will also be examined across provinces and hospital levels. Fourth, the use of a self‐administered retrospective questionnaire may have introduced recall bias, social desirability bias, and measurement variability. Although anonymity was ensured and managers were trained to provide neutral clarification, perceived hierarchical pressure cannot be completely excluded. Future studies should incorporate multi‐source data and standardized WPV reporting systems. Finally, the included variables did not cover patient‐, family‐, and event‐level characteristics. Future studies should incorporate more comprehensive multilevel information to improve model generalizability.

## 6. Conclusions

This study developed and validated a primary nomogram‐based model using the original data to stratify the concurrent risk of moderate‐to‐high frequency WPV among Chinese pediatric nurses, with SMOTE‐based analysis as a complementary approach. The primary model showed stable discrimination and acceptable calibration, suggesting its potential utility as a practical risk‐stratification tool in the study population. Hospital level, nurse–child–caregiver communication conflicts, tolerance of violence, occupational stress, and burnout were independently associated with moderate‐to‐high frequency WPV, suggesting that individual, interpersonal, organizational, and policy factors should be considered in WPV prevention. The SMOTE‐based analysis provided complementary information on the influence of class imbalance and showed higher sensitivity, but it had poorer calibration in the original test set. The web‐based calculator may support individualized risk estimation and help nursing managers identify nurses who may benefit from targeted preventive measures, such as communication training, stress management, and improved institutional response mechanisms. Given the cross‐sectional design and lack of external validation, further prospective, multicenter validation studies are needed to clarify temporal relationships, evaluate model transportability, and assess the practical value of the nomograms in routine nursing management.

## 7. Implications for Nursing Management

The findings of this study have several implications for nursing management. First, the nomogram and web‐based calculator, as decision‐support tools, may help nursing managers identify pediatric nurses with a higher estimated probability of recent moderate‐to‐high frequency WPV exposure and support structured risk stratification. In practice, the threshold interval of 5%–34% may inform threshold selection for WPV risk stratification, with the primary model cutoff of 19.5% serving as a candidate operating threshold. Threshold selection should balance the consequences of false‐negative and false‐positive classifications while considering the resource requirements and potential burden of subsequent assessment and support measures. Lower thresholds may be appropriate when minimizing missed cases is prioritized and such measures require limited resources and impose minimal burden, whereas higher thresholds may be considered when these measures impose substantial burdens on nurses or institutions. Second, risk stratification may help prioritize preventive resources for nurses classified as high risk. Relevant measures may include communication and de‐escalation training, timely reporting channels, psychological support, stress management, and burnout reduction programs. These interventions may be integrated into a multicomponent WPV prevention strategy rather than delivered as isolated training activities. Third, these findings suggest that individual‐level interventions alone may not adequately address the multidimensional nature of WPV risk and that organizational‐level measures should be addressed in parallel. Nursing managers may consider strengthening organizational measures, including clear reporting procedures, rapid response mechanisms, nonpunitive support for affected staff, regular review of staffing and workflow arrangements, and accountability mechanisms for handling WPV incidents. Finally, the findings may inform the refinement of hospital‐level occupational safety policies and WPV management systems within healthcare institutions.

## Author Contributions

Xuemei Ding analyzed data and drafted the paper, Huichen Zhang analyzed data and revised the paper, Mengting Liu drafted the paper, Qingmei Ding and Xiaoyu Pan collected data, and Yuhong Zhang and Xiaoli Zhang designed and supervised the paper.

## Funding

The work was supported by the Continuing Education Teaching Reform and Research Project of Binzhou Medical University (Grant Number: BYJJ202401), the Shandong Provincial Social Science Planning Research Project (Grant Number: 23CSHJ01), and the Graduate Education Reform and Quality Improvement Project of Shandong Province (Grant Number: SDYJG19163).

## Disclosure

The funders are not involved in any aspect of the research, including study design, data collection and analysis, manuscript writing, or its submission. All the authors approved the submitted paper.

## Ethics Statement

Ethics approval was given by the Binzhou Medical University, reference number 2021‐352.

## Conflicts of Interest

The authors declare no conflicts of interest.

## Supporting Information

Additional supporting information can be found online in the Supporting Information section.

## Supporting information


**Supporting Information** Supporting File 1 is the TRIPOD + AI checklist. Supporting File 2 presents a comparison of the multivariable logistic regression models between the primary analysis and the MICE analysis. Supporting File 3 shows the sensitivity analysis evaluating the impact of adding work experience to the primary model on model estimates.

## Data Availability

The data that support the findings of this study are available on request from the corresponding author. The data are not publicly available due to privacy or ethical restrictions. Upon approval, the research team will also provide the trained statistical model files implemented in R, facilitating replication and further scientific inquiry.
