# Supplementary material for: Development and Validation of a Nomogram‐Based Risk Stratification Model for Moderate‐to‐High Frequency Workplace Violence Among Chinese Pediatric Nurses: A Cross‐Sectional Study
Source: J Nurs Manag. 2026 Sep 24;2026:6562210. doi: 10.1155/jonm/6562210 (PMC13602356; doi:10.1155/jonm/6562210)
Supplement: Supplementary file 1 [file JONM-2026-6562210-s001.zip › Supplementary File 2.docx]

Supplementary File 2. Comparison of multivariable logistic regression models between the primary analysis and the MICE

| Factors | Primary analysis | | MICE | |
| --- | --- | --- | --- | --- |
|  | OR (95% CI) | *P* | OR (95% CI) | *P* |
| Self-perceived health status (ref: good) | | | | |
| Fair | 1.737(0.865–3.487) | 0.120 | 1.645(0.817–3.313) | 0.164 |
| Poor | 1.948(0.828–4.581) | 0.126 | 1.928(0.818–4.544) | 0.133 |
| Hospital level (ref: class III grade B hospital) | | | | |
| Class III grade A hospital | 0.513(0.273–0.967) | **0.039** | 0.515(0.273–0.971) | **0.040** |
| Nurse-child-caregiver communication conflicts (ref: no) | | | | |
| Yes | 3.140(1.705–5.786) | **<0.001** | 3.121(1.692–5.756) | **<0.001** |
| Hospital attitudes to WPV (ref: proactive protection of employee rights) | | | | |
| Delayed responses | 1.914(0.685–5.346) | 0.215 | 1.947(0.695–5.449) | 0.205 |
| Tolerance of violence | 3.784(1.102–12.992) | **0.034** | 3.953(1.147–13.615) | **0.029** |
| Blaming employees | 1.985(0.606–6.506) | 0.258 | 1.940(0.591–6.365) | 0.274 |
| Occupational stress | 1.034(1. 010–1.059) | **0.005** | 1.036(1.012–1.061) | **0.004** |
| Burnout (ref: no) | | | | |
| Yes | 8.513(1.111–65.210) | **0.039** | 8.638(1.128–66.156) | **0.038** |
