# Supplementary material for: Development and Validation of a Nomogram‐Based Risk Stratification Model for Moderate‐to‐High Frequency Workplace Violence Among Chinese Pediatric Nurses: A Cross‐Sectional Study
Source: J Nurs Manag. 2026 Sep 24;2026:6562210. doi: 10.1155/jonm/6562210 (PMC13602356; doi:10.1155/jonm/6562210)
Supplement: Supplementary file 1 [file JONM-2026-6562210-s001.zip › Supplementary File 3.docx]

Supplementary File 3. Sensitivity analysis evaluating the impact of adding working experience on model estimates

| Variables | Primary analysis | | Adjusted model | | Change in *β* |
| --- | --- | --- | --- | --- | --- |
|  | *β* | OR (95% CI) | *β* | OR (95% CI) |  |
| Self-perceived health status (ref: good) | | | | | |
| Fair | 0.552 | 1.737(0.865–3.487) | 0.550 | 1.733 (0.862, 3.484) | -0.002 |
| Poor | 0.667 | 1.948(0.828–4.581) | 0.650 | 1.916 (0.813, 4.520) | -0.017 |
| Hospital level (ref: class III grade B hospital) | | |  |  |  |
| Class III grade A hospital | -0.667 | 0.513(0.273–0.967) | -0.692 | 0.501 (0.262, 0.956) | -0.025 |
| Nurse-child-caregiver communication conflicts (ref: no) | | | | | |
| Yes | 1.144 | 3.140(1.705–5.786) | 1.202 | 3.326 (1.787, 6.190) | +0.058 |
| Hospital attitudes to WPV (ref: proactive protection of employee rights) | | | | | |
| Delayed responses | 0.649 | 1.914(0.685–5.346) | 0.666 | 1.946 (0.693, 5.468) | +0.017 |
| Tolerance of violence | 1.331 | 3.784(1.102–12.992) | 1.363 | 3.910 (1.127, 13.559) | +0.032 |
| Blaming employees | 0.686 | 1.985(0.606–6.506) | 0.671 | 1.956 (0.594, 6.442) | -0.015 |
| Occupational stress | 0.034 | 1.034(1. 010–1.059) | 0.033 | 1.034 (1.009, 1.059) | -0.001 |
| Burnout (ref: no) | | | | | |
| Yes | 2.142 | 8.513(1.111–65.210) | 2.095 | 8.125 (1.062, 62.180) | -0.047 |
| Work experience (ref: <5 years) | | | | | |
| 5–10 years | **-**  **-** | - | 0.155 | 1.167 (0.548, 2.488) | - |
| >10 years |  | - | 0.508 | 1.662 (0.802, 3.444) | - |
